# The CD73/Ado System—A New Player in RT Induced Adverse Late Effects

**DOI:** 10.3390/cancers11101578

**Published:** 2019-10-16

**Authors:** Simone de Leve, Florian Wirsdörfer, Verena Jendrossek

**Affiliations:** Institute of Cell Biology (Cancer Research), University Hospital Essen, 45122 Essen, Germany; simone.deleve@uk-essen.de (S.d.L.); florian.wirsdoerfer@uk-essen.de (F.W.)

**Keywords:** purinergic signaling, radiotherapy, normal tissue toxicity, pneumonitis, fibrosis, adenosine, tumor microenvironment, therapeutic window

## Abstract

Radiotherapy (RT) is a central component of standard treatment for many cancer patients. RT alone or in multimodal treatment strategies has a documented contribution to enhanced local control and overall survival of cancer patients, and cancer cure. Clinical RT aims at maximizing tumor control, while minimizing the risk for RT-induced adverse late effects. However, acute and late toxicities of IR in normal tissues are still important biological barriers to successful RT: While curative RT may not be tolerable, sub-optimal tolerable RT doses will lead to fatal outcomes by local recurrence or metastatic disease, even when accepting adverse normal tissue effects that decrease the quality of life of irradiated cancer patients. Technical improvements in treatment planning and the increasing use of particle therapy have allowed for a more accurate delivery of IR to the tumor volume and have thereby helped to improve the safety profile of RT for many solid tumors. With these technical and physical strategies reaching their natural limits, current research for improving the therapeutic gain of RT focuses on innovative biological concepts that either selectively limit the adverse effects of RT in normal tissues without protecting the tumor or specifically increase the radiosensitivity of the tumor tissue without enhancing the risk of normal tissue complications. The biology-based optimization of RT requires the identification of biological factors that are linked to differential radiosensitivity of normal or tumor tissues, and are amenable to therapeutic targeting. Extracellular adenosine is an endogenous mediator critical to the maintenance of homeostasis in various tissues. Adenosine is either released from stressed or injured cells or generated from extracellular adenine nucleotides by the concerted action of the ectoenzymes ectoapyrase (CD39) and 5′ ectonucleotidase (NT5E, CD73) that catabolize ATP to adenosine. Recent work revealed a role of the immunoregulatory CD73/adenosine system in radiation-induced fibrotic disease in normal tissues suggesting a potential use as novel therapeutic target for normal tissue protection. The present review summarizes relevant findings on the pathologic roles of CD73 and adenosine in radiation-induced fibrosis in different organs (lung, skin, gut, and kidney) that have been obtained in preclinical models and proposes a refined model of radiation-induced normal tissue toxicity including the disease-promoting effects of radiation-induced activation of CD73/adenosine signaling in the irradiated tissue environment. However, expression and activity of the CD73/adenosine system in the tumor environment has also been linked to increased tumor growth and tumor immune escape, at least in preclinical models. Therefore, we will discuss the use of pharmacologic inhibition of CD73/adenosine-signaling as a promising strategy for improving the therapeutic gain of RT by targeting both, malignant tumor growth and adverse late effects of RT with a focus on fibrotic disease. The consideration of the therapeutic window is particularly important in view of the increasing use of RT in combination with various molecularly targeted agents and immunotherapy to enhance the tumor radiation response, as such combinations may result in increased or novel toxicities, as well as the increasing number of cancer survivors.

## 1. Background

Current calculations from the National Cancer Institute estimate that 38.4% of the human population worldwide will be diagnosed with cancer during their lifetime and that until 2030, the number of newly diagnosed cancer patients per year will rise to 23.6 [1]. Despite constant improvements in cancer treatment and decreasing death rates, cancer is still a life-threatening disease: 9.6 million cancer patients worldwide died from their disease in 2018, and 20.3% of these deaths occurred in Europe [2] highlighting the need for further improvements in cancer therapy.

Together with surgery and chemotherapy radiotherapy (RT) belongs to the current three standard treatment options for cancer patients. More than 60% of all cancer patients receive RT at least once during the course of their disease with curative or palliative intent [3,4,5]. Though RT has a documented contribution to enhanced local control and overall survival of cancer patients and cancer cure, patient outcome needs to be further improved for common forms of cancer with high loco-regional failure-rates or frequent development of metastases. Avoiding adverse effects in normal tissues is another major challenge in clinical RT, particularly in tumors growing adjacent to critical structures or within tissues or organs with pronounced radiation sensitivity.

In fact, acute and late toxicity to normal tissues at risk is an important biological barrier to be successful in RT: normal tissue toxicity precludes the use of curative RT doses and may thus result in suboptimal local tumor control or metastatic disease even when accepting side-effects that decrease quality of life [3,6,7]. Furthermore, normal tissue toxicity limits therapy intensification efforts for many locally advanced tumors through the combination RT with cytotoxic chemotherapy [8,9,10]. Documented toxic effects of RT include for example acute toxicity to the hematopoietic system, gastrointestinal tract, skin, or mucosal tissues (mucositis, pneumonitis); acute toxic effects are mostly reversible, but can be life-threatening when they are severe. Instead, long-term adverse effects such as disturbance of proper bone growth, fibrotic lung disease, cardiotoxicity, cognitive impairment, as well as induction of secondary tumors are considered to be mostly irreversible and progressive, and can thereby strongly impact the quality of life of the patients, as well as patient survival.

A certain RT dose is tolerable if the probability of an effective tumor response is higher than the probability of significant adverse effects to critical normal tissues within or adjacent to the radiation field. Improvements in the therapeutic gain of RT, can thus be obtained by enhancing the precision of RT delivery, as well as from biological strategies interfering with molecular or cellular mechanisms driving tumor or normal tissue responses to RT. Herein, RT practice has largely benefited from technical improvements in treatment planning, e.g., stereotactic radiotherapy or intensity-modulated radiation therapy, that increased accuracy of dose delivery while reducing normal tissues exposure. Furthermore, therapy approaches with charged particles (mainly protons, and carbon ions) deliver only a limited amount of energy to superficial tissues while allowing the deposition of high radiation doses with high accuracy to deep-seated tumors [11,12,13]. Advances in dose delivery to improve tumor killing with minimal toxicity have also been described using ultrahigh dose-rate (FLASH) irradiation with rates above 100Gy/sec [14,15,16,17]. For example, a recent case report revealed promising results regarding safety and outcome both on normal tissue and tumor [18]. Clinical translation of emerging FLASH radiotherapy has been discussed in recent reviews highlighting the need of further studies and technical improvements in this aspiring research field [19,20]. However, some patients still suffer from adverse effects highlighting the high medical need for further improvements. 

Beyond dose-limiting normal tissue toxicity, further biological factors limit the efficacy of RT and thereby decrease the life quality of the treated patients [11]: These include amongst others intrinsic and environment-induced radioresistance of tumor cells, individual heterogeneity in tumor and normal tissue responses to IR, phenotypic plasticity of cancer cells, as well as enrichment in radioresistant cancer stem cells [21,22,23,24,25,26,27,28,29]. Further improvements in the outcome of patients with locally advanced tumors are therefore expected from treatment protocols combining precision RT with mechanism-based drug-therapy [5,30]. Recent progress in immunotherapy and new insight into the biologic effects of IR suggest that the combination of highly conformal RT with immunotherapy may also be suited to further improve therapy outcome [31,32].

However, preclinical and clinical investigations demonstrate that toxicity rates can increase or new toxicities can occur when using molecularly targeted drugs such as angiogenesis inhibitors, DNA repair pathway blockers or immune checkpoint inhibitors in combination with RT as part of a biologically optimized RT [11,33,34,35,36]. Thus, it will be favorable to combine RT with mechanism-based drug therapy suited to attenuate or mitigate adverse late effects or protect normal tissues from the toxic effects of RT if we aim to design effective strategies for improving the therapeutic gain of RT and enhance cure rates in combinatorial approaches [37].

Exposure to IR damages cellular macromolecules, e.g., proteins, lipids, as well as nuclear and mitochondrial DNA, either directly or indirectly though induction of reactive oxygen species. Induction of double strand breaks (DSB) in genomic DNA is considered as most lethal event; therefore, it is not surprising that cells have developed a multifaceted DNA damage response (DDR) that is initiated within minutes upon irradiation and reflects the severity and the biology of the induced DNA damage (for detailed review of the DDR please refer to [38,39,40]).

Local irradiation of normal tissues usually hits several different resident cell types, e.g., epithelial cells, endothelial cells as well as tissue stem cells, so that the resulting response will reflect the integration of the DDR of the affected cell types. The resulting cellular responses to IR initiate a complex cascade of events that can cause acute toxicity, chronic adverse effects, or both [41,42,43,44].

Cellular responses to RT-induced damage or cell death trigger a cascade of events that leads within days or weeks to activation of innate and adaptive immune responses, pronounced inflammation (e.g., skin toxicity, mucositis, pneumonitis), vascular damage or dysfunction, or after months to excessive deposition of extracellular matrix molecules and tissue scarring (fibrosis) with potential impact on the quality of life and patient survival [45,46]. Indeed, deregulated cytokine production and infiltration of immune cells are frequent pathologic findings in irradiated tissues and are also observed in murine models [45,47,48,49,50,51,52,53,54,55]. Analyses in preclinical models and patients’ samples demonstrate a complex tissue response with multiple interactions between resident cells, stromal factors such as vascular cells, stromal fibroblasts and tissue stem cells, and infiltrating immune cells [46,56,57].

Though RT exerts early immunosuppressive effects by efficient killing of immune cells within the radiation field [58], acute tissue responses to RT clearly involve activation of various aspects of innate and adaptive immune responses that have been extensively reviewed elsewhere [59,60,61,62,63,64,65,66]. Importantly, immune responses to RT have a dual face as they may induce the accumulation of immunoregulatory or even immunosuppressive mediators and immune cell types with tumor-promoting characteristics [42,67,68,69] that are reminiscent of processes involved in tumor immune escape [70,71,72]. Adverse inflammatory processes in irradiated normal tissues frequently respond to steroid therapy. Nevertheless, it is still controversial if infiltrating immune cells and endogenous regulators of tissue inflammation and inflammatory resolution directly contribute to the pathophysiology of RT-induced adverse late effects or only modulate disease progression [73].

Herein, recent reports including own findings highlight a pathogenic role of the immunoregulatory ecto-5′-nucleotidase (NT5E, CD73)/adenosine system in radiation-induced adverse late effects [68,74,75]. Generally, adenosinergic signaling is an essential endogenous regulator of tissue homeostasis that is involved in maintenance and reconstitution of tissue homeostasis upon stress or injury [76,77,78,79].

Thus far, there are only limited investigations about the role of CD73 and adenosine in RT-induced normal tissue toxicity. Here, we summarize current knowledge on the role of CD73 and adenosine in promoting radiation-induced normal tissue toxicity with a special focus on its general and tissue-specific contribution to IR-induced fibrotic disease. We will also highlight pathologic aspects of CD73/adenosine signaling in malignant tumors and discuss the potential benefit of CD73/adenosine targeting strategies for improving the therapeutic gain of RT.

## 2. Purinergic Signaling and Tissue Homeostasis

CD73 and adenosine are critical endogenous regulators of tissue homeostasis balancing tissue inflammation and repair processes in diverse pathological situations and preventing autoimmunity [78,80,81,82]. Upon controlled (Pannexin 1 channel) or uncontrolled release into the extracellular room ATP acts as a danger signal that initiates pro-inflammatory signaling cascades through binding to P2 receptors (the ionotropic P2X receptors or the metabotropic P2Y receptors) [83,84,85,86,87]. Extracellular ATP is usually rapidly converted to ADP and AMP via membrane bound ectoenzymes of the ectonucleoside triphosphate diphosphohydrolases (ENTPDases) or alternatively nucleotide pyrophosphatase/phosphodiesterases (NPPases) that thereby terminate ATP signaling through P2X or PTY nucleotide receptors [80,82,88].

**Figure 1:**
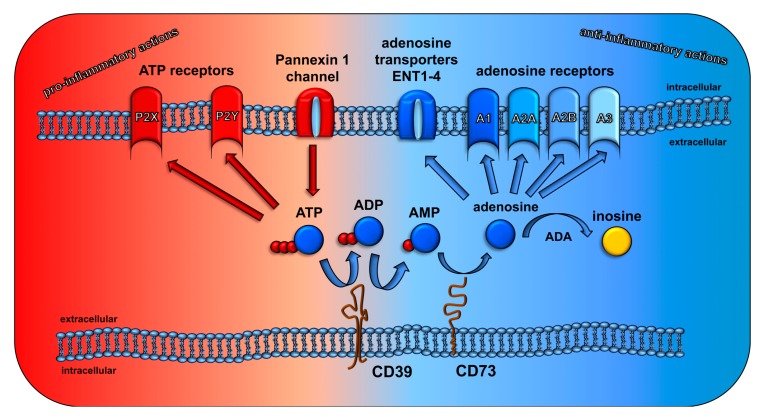
Purinergic Signaling. ATP can be released uncontrolled into the extracellular room by damaged or dead cells or actively via Pannexin1 channels. If ATP is released into the extracellular room multiple processes can take place. It can bind to different ATP receptors (P2 receptors) and in consequence pro-inflammatory signaling cascades will be induced. On the other hand, extracellular ATP can be rapidly converted to adenosine via two membrane bound ecto-nucleotidases CD39 and CD73. Binding to one of four different receptors adenosine exerts mostly immunoregulatory effects. Alternatively, extracellular adenosine can be shuttled into target cells via adenosine transporters or is converted via the adenosine deaminase to inosine.

CD39 (ectonucleoside triphosphate diphosphohydrolase1, ENTPD1) is mainly expressed on the surface of various immune cells but also on epithelial, endothelial cells and specific subpopulations of fibroblasts as described elsewhere [89,90,91,92,93,94]. CD39 is responsible for ATP degradation into ADP and further into AMP that will fuel CD73 activity and the generation of adenosine (Figure 1). CD73 acts in concert with CD39 to generate adenosine from extracellular AMP [81,95]. CD73 is found on the surface of various cell types, particularly endothelial cells [84] and immune cells, e.g., conventional CD4 and CD8 T cells, murine regulatory T cells (T_reg_), regulatory B cells (B_reg_) and macrophages, but also mesenchymal stromal cells (MSC) and epithelial cells from various healthy tissues [82,91,92,96,97,98,99,100,101,102]. As mentioned above several murine immune cells as well as resident cells express CD39 and CD73. Of note, the expression patterns of CD39 and CD73 differ between mice and humans as summarized by Allard and colleagues in detail [103]. Briefly, in human peripheral blood CD39 is constitutively expressed on 20–30% of CD4^+^ T cells (including memory T cells and T_reg_), <5% of CD8^+^ T cells, 2–5% of NK cells, >90% of B cells and >90% of monocytes. The expression of CD73 in human peripheral blood is 10% of CD4^+^ T cells, 50% of CD8^+^ T cells, 75% of B cells and 2–5% of NK cells.

The major discrepancy between mice and humans is that in humans, only 1–5% of T_reg_ express surface-bound CD73. Compared to conventional T cells, human T_reg_ show however enriched expression (75%) of intracellular CD73 [104]. Moreover, several recent studies highlight that human T_reg_ do not co-express CD39 and CD73 on their surface but instead need to cooperate with other cells expressing CD73, to generate adenosine and mediate immunosuppression [105,106,107]. Studies investigating the expression of CD73 on T cells subsets in humans are rare, but Doherty and colleagues suggest a potential link between a Th17 profile and CD73 expression on memory CD4^+^ T cells in patients with inflammatory bowel disease [108]. Similar results were obtained by Schuler and co-workers demonstrating that human CD4^+^ CD73^+^ T cells belong to a subset of T cells expressing the memory marker CD45RO as well as the activation marker CD26 [109]. In line with the findings described above, Gourdin et al. reported, that CD73 expression defined a subset of polyfunctional Th1.17 cells that infiltrated breast and ovarian tumors. Moreover, CD39^+^ T_reg_ inhibited these CD73^+^ Th1.17 cells through cooperative degradation of ATP into adenosine [105]. Regarding the role of CD73 on CD8^+^ T cells Bono and coworkers reported that CD73 expression is high in naïve CD8^+^ T cells and is down-regulated upon activation. The authors suggest that CD73 down-regulation prevents autocrine adenosine signaling leading to successful differentiation into effector T cells [110]. As mentioned above, CD39 and CD73 are highly expressed on human B cells. Moreover, a study from Schena et al showed, that human B cells can store ATP in secretory vesicles that can be released upon B cell receptor activation. Furthermore, secreted ATP can be hydrolyzed into adenosine in an autocrine manner via CD39 and CD73 on these B cells which is important for class switch recombination [111]. The expression of CD73 on human macrophages can differ regarding the macrophage phenotype but is controversially discussed. Zanin et al. revealed that human pro-inflammatory M1 macrophages express lower levels of CD39 and CD73 compared to anti-inflammatory M2 macrophages [112]. In contrast, findings from Eichin and colleagues highlight that CD73 expression on human macrophages is induced after incubation with the LPS and TNF-α, relevant for in vitro M1 macrophage polarization [113].

Thus, the distinct CD73 expression patterns on human immune cell subsets could impact the use of pharmacologic inhibition of CD73/adenosine signaling in human pathologies when compared to mice and these potential differences need further investigation.

In contrast to ATP, adenosine has mostly immuno-regulatory actions and triggers anti-inflammatory responses to limit inflammation-induced tissue damage [82,114]. Furthermore, CD73 and adenosine not only regulate the extravasation and function of diverse immune cells but also modulate epithelial cell behavior, vascular barrier function, and cell death [81,98,99,115,116,117,118,119,120]. The extracellular level of adenosine in normal tissues is usually low (10–100 nM) [121,122] due to its very short half-life, as there exists a rapid cellular uptake by nucleoside transporters (equilibrative nucleoside transporters (ENT) and concentrative nucleoside transporters (CNT)) as well as extracellular or intracellular catabolic conversion by adenosine deaminase (ADA) or adenosine kinase (ADK) to inosine or AMP, respectively [80,123]. This strongly suggests that adenosine is a local modulator [124,125] and might only act on cells in narrow environment near CD73 expressing cells. However, adenosine levels can rapidly increase in response to stress, hypoxia, or tissue injury either by direct release from damaged cells or through ATP/ADP catabolism by CD39 and CD73 reaching µmolar levels [121,122,126,127]. Using gradient ion-repair, reversed-phase HPLC at 254 nm [128], Vaupel and colleagues report tumor adenosine levels between 50 and 90 µM in experimental DS-sarcoma in rats with rising concentrations in tumors with increasing hypoxic fractions or tumor volume, respectively [126,127]. Instead, Blay and colleagues report only mean steady state concentrations of 0.3–0.5 µM adenosine in extracellular tumor fluid of murine and human colon (MCA-38, T-84, HT-29) and human A549 lung carcinomas in mice. These levels increased to 9–13 µM upon pharmacologic inhibition of adenosine degradation [122]. Finally, data from the Sitkovsky group [121] revealed higher levels of adenosine (about 0.2–1.3 µM) in MCA-205 fibrosarcoma in mice that were associated with pronounced hypoxia. Nevertheless, levels of extracellular adenosine in experimental tumors were always significantly higher than the adenosine levels in normal tissues that were in the nM range (30 ± 5 nM or below 50 nM [121,122] in skin tissue, as well as 100 nM and 300 nM in bronchoalveolar lavage fluid of murine lungs without or with exposure to whole thorax irradiation [74], respectively.

Extracellular adenosine can bind to and act through one of four different adenosine receptors (ADOR) A1, A2A, A2B and A3. Various immune cells among them neutrophils, monocytes, macrophages and lymphocytes express one or several of the 4 different G-protein-coupled adenosine receptors (ADORA1, ADORA2A, ADORA2B, ADORA3) on their cell surface [90,129,130,131,132,133,134,135,136,137,138,139]. While the activation of ADORA1 and ADORA3 lead to a decrease in intracellular cAMP concentrations as they are coupled to adenylate cyclase via the Gi/o subunits, coupling through the Gs subunit in ADORA2A and ADORA2B results in increased cAMP levels [80,140]. Under physiological conditions extracellular adenosine levels are below 1 µM but high enough to activate ADORA1, ADORA2A and ADORA3 receptors. For the activation of ADORA2B adenosine levels higher than 10 µM are needed, which are only achieved under stress conditions or pathophysiological conditions [123,126].

Of note, while acute activation of CD73 and adenosine mostly exert tissue-protective effects, chronic extracellular accumulation of adenosine has widely been associated with pathologic processes, for example the development of tissue fibrosis e.g., in the skin, the lung, the liver, and the heart [74,78,141,142,143,144].

## 3. Modeling Radiation-Induced Adverse Late Effects In Vivo

Various tissues (e.g., skin, gut, lung, kidney, liver) are sensitive to late adverse radiation effects such as vascular injury, inflammation, necrosis, fibrosis, and tissue dysfunction [145]. Particularly the progressive late adverse effects limit the total dose that can be safely applied during the treatment [43,146].

Radiation-induced acute and chronic toxicities involve complex interactions between various types of resident cells, soluble mediators, infiltrating immune cells, and extracellular matrix molecules. Except from the type of tissue, the pathophysiology of the observed toxicities depends on additional factors, e.g., dose, treatment schedule, type and location of irradiation, as well as the functional state of the organ at risk at the time of irradiation [7]. In vitro model systems with co-culture of two or three different cell types have thus only limited power to predict the ongoing processes in the human body so that it is unavoidable to use in vivo model systems to study radiation-induced toxicities.

Different approaches are used to study radiation-induced adverse normal tissue effects in preclinical investigations in vivo: Many laboratories use preclinical rodent models (mostly mice or rats) to gain insight into potential effector cells and involved mediators [51,52,53,54,56,74,147,148,149,150,151,152,153,154,155,156]. Therefore, defined regions of the animals are exposed to photon irradiation using different sources (x-Rays, Co^60^, linear accelerator) and either single high dose irradiation or fractionated irradiation schemes [46,51,53,68,156,157,158]; further variations include the use of different radiation qualities, e.g., irradiation with photons versus protons [159,160].

In some studies the antibiotic chemotherapeutic agent bleomycin is used as a surrogate for exposure to IR. Bleomycin induces single and double strand breaks in the DNA and is thus considered as useful to mimic irradiation-induced damage [161]. However, the instant action of IR and the protracted action of bleomycin will result in differences in the biology of the resulting DNA damage. Furthermore, bleomycin-induced normal tissue damage in vivo largely depends on the administration route and treatment schedule resulting in prominent differences in the tissue and immune responses induced by local versus systemic administration [162,163,164]. Therefore, we consider bleomycin as a surrogate with a limited translational capacity.

## 4. Tissue-Specific Radiotoxicities

Preclinical investigations of damage-induced sterile inflammation and fibrotic disease revealed that despite disease-specific mechanisms and pathologic drivers, the different pathologies share some common hallmarks reminiscent of wound healing processes [42,165]. Moreover, pulmonary fibrosis induced as an adverse late effect of RT, chemotherapy and some targeted agents has pathophysiologic features of idiopathic pulmonary fibrosis [46,166]. In vivo models of tissue fibrosis and the use of genetically modified mice have advanced our understanding of the underlying cellular and molecular mechanisms and helped to identify signaling molecules that participate in the pathogenesis of adverse late effects induced by exposure to IR in normal tissues, particularly the lung [45,46,56,57].

### 4.1. Lung

Patients suffering from malignant neoplasms in the thoracic region e.g., lung, breast or head, and neck cancer, as well as patients who receive total body irradiation (TBI) in conditioning regimens for stem cell or bone marrow transplantations may receive a certain radiation dose to the highly radiosensitive lung tissue [41].

Depending on the radiation dose and the irradiated lung volume receiving ≥ 20 Gy, sensitive patients (5–20%; > 30% for patients receiving TBI) typically develop pneumonitis at 2–6 months whereas pulmonary fibrosis is observed 6 to 24 months after radiotherapy and may become chronic in patients with a larger irradiated lung volume [7,45]. Interestingly, early investigations reported a correlation of pneumonitis with the presence of increased levels of CD4^+^ T-lymphocytes in the bronchioalveolar lavage fluid (BALF) of irradiated breast or lung cancer patients [167,168] whereas the predictive value of cytokine plasma levels for radiation pneumonitis remains controversial [169,170]. The influx of immune cells e.g., lymphocytes, monocytes, neutrophils, and macrophages and associated changes in the cytokine and chemokine levels also characterizes this acute and chronic phases of lung injury in preclinical models [47,48,49,51,56,68,169]. Up to now, no causative but only symptomatic treatment with glucocorticoids is available, aimed at reducing inflammation and attenuating endothelial cell toxicity during the pneumonitic phase [171,172,173]. Other anti-inflammatory agents (Azathioprine and cyclosporine) were shown to effectively dampen the symptoms of pneumonitis and may be used if glucocorticoids are not suitable [171,174].

Also no causative therapy for prevention or treatment of radiation-induced lung fibrosis (RILF) is available in the clinic, though several approaches are under experimental investigation [43,175,176].

Currently, researchers mostly use single high dose whole thorax or hemithorax irradiation of fibrosis-sensitive C57BL/6 mice to gain insight into disease pathogenesis as this strain is reminiscent of the clinical course of the disease and allows functional investigations about the role of identified factors using knockout strains with deficiency of the gene of interest [51,53,68,74,158,162]. However fibrosis sensitive C57BL/6 mice develop only mild pneumonitis; therefore, thoracic irradiation of C57L mice or regional irradiation of rat lungs might be better suited for investigations of pneumonitis and lung fibrosis, respectively [56,158,162,177].

During recent years various mediators have been identified that participate in the pathogenesis of radiation-induced lung disease (RILD), such as transforming growth factor β1 (TGF-β), platelet-derived growth factor (PDGF), connective tissue-derived growth factor, interleukin-6 (IL-6), IL-10, death receptor CD95 and its ligand, surfactant protein-A (SP-A) and SP-D, nuclear factor erythroid 2–related factor 2 (Nrf-2) but molecularly-targeted drugs did not make it yet to the clinics [7,45,47,56,57,172,178,179,180,181].

### 4.2. Skin

Radiation-induced damage to the skin is a major problem in cancer therapy as 90–95% of all radiation-treated individuals develop some degree of skin toxicity within the irradiated field, at least when photon irradiation is used [182,183]. First signs of acute skin toxicity can usually be detected within the first four weeks after RT start. The effects are cumulative, and once manifested, stay throughout the treatment and last 2–4 weeks after end of therapy [182,184,185]. Acute dermal radiation injury, also called radiation dermatitis, is characterized by erythema, dry/moist desquamation up to ulceration depending on its grade [184,185].

Instead, chronic radiation dermatitis observed months to years after RT is rare, and includes skin atrophy, over hypo-/hyperpigmentation, ulcerations and fibrosis [183,184,186]. Treatment of radiation-induced dermal injuries includes standard wound management and hygiene, adequate creams, and antibiotics [185]. Furthermore, treatment with pentoxifylline/alpha-Tocopherol and hyperbaric oxygen have shown promising results [187,188]. Tissue transfer to reconstruct irradiated areas has also shown good results as long as the patient’s vessels are still accessible in the fibrotic tissue [188]. 

Interestingly, acute and chronic dermal injuries in irradiated patients are also characterized by immune cell infiltrates [182,189]. Furthermore, patient data revealed high levels of pro-inflammatory cytokines like TNF-α, IL-6 and IL-1, as well as pro-fibrotic drivers like TGF-β, PDGF and CTGF that are thought to promote pathogenesis by activating fibroblasts, inducing ECM secretion and recruitment of further leukocytes [183].

Various murine models exist to study radiation-induced skin toxicity that vary in dose, fractionation, as well as size and location of the irradiation field [147,148,149]. Single dose irradiation models are common and are used by several groups [75,147,148,149,190]. Of note, infiltration of immune cells is also a common feature of radiation-induced skin toxicity in mice though the infiltration-intensity is strain specific [149].

### 4.3. Gut

Radiation toxicity to the gastrointestinal tract is also highly dependent on the applied dose per fraction and the fractionation schedule as well as on the irradiated volume [191,192]. Up to 90% of the irradiated patients present changes in the bowl habits and 50% of those experience a reduced quality of life after RT [193]. Early symptoms of radiation injury occur during or up to 90 days after therapy and include acute mucosal injury and inflammation [191]. Anti-inflammatory agents like five aminosalicylic acid (5-ASA) are suggested to reduce acute side effects of irradiation, but up to now a standardized use is not investigated sufficiently [194]. Acute tissue responses are furthermore characterized by the occurrence of growth factors, inflammatory mediators, and cytokine cascades [150,191]. In severe inflammation, mediators secreted from infiltrated monocytes, macrophages and granulocytes promote subsequent tissue damage, epithelial cell loss and degradation of ECM, which in turn may result in tissue fibrosis [195].

Chronic intestinal radiation injury occurs months or years following radiotherapy and is accompanied by side effects like fibrosis, vascular sclerosis, diarrhea, abdominal or bottom pain, fecal incontinence and intestinal blood loss [191,193,196]. There is one study showing a protection of chronic irradiation side effects using antioxidants like vitamin E and C, whereas investigations using Amifostine or Sucralfate still lack sound data to recommend usage in the clinical routines [194]. Nevertheless, several agents with assumed protective action are under current pre-clinical investigations.

Animal models to study the pathology of radiation-induced intestinal injury range from whole body or abdominal irradiation models up to irradiation models that include surgical exteriorization of the small intestine or models where only parts of the intestine can be irradiated [150,151,197]. In addition, a recent report introduced an image-guided murine model, which better simulates the situation observed in the clinics and is such suitable for the analysis of potential radioprotectors [150].

### 4.4. Kidney

Radiation-induced damage to the kidneys can also be subdivided into acute and chronic nephropathy, where acute injuries are detected within three months after RT and chronic side effects are usually detected from 18 months following therapy [198,199]. Characteristic symptoms of renal radiation injury are reduced glomerular filtration rate, anemia, hypertension, edema and renal failure [198,199,200]. The kidneys are extremely sensitive to irradiation as it was shown that doses of 4 Gy can cause renal injury, thereby limiting the applied dose for example for TBI [198,200].

So far, only limited preclinical investigations about radiation-induced kidney toxicity are available. Due to the importance of renal toxicity in nuclear medicine applications most preclinical studies focus on toxicity of radiopharmaceuticals like ^131^I, ^211^At, and ^177^Lu on normal renal tissues [201,202,203,204,205]. Furthermore, some preclinical studies used irradiation models with X-rays, e.g., exposure of the right kidney to a single dose of 8–10 Gy [206]. More common are experiments with exposure to TBI where the normal tissue response of the kidneys is studied among other organs [207,208]. So far, no data are available about the importance of radiation-induced immune changes in kidney injury. However, in the model of unilateral ureteral obstruction (UUO)-induced renal fibrosis it has been reported that macrophages infiltrate the injured kidney and change their phenotype from pro-inflammatory during the acute damage to anti-inflammatory during fibrosis development [209,210]. This suggests, that damage-induced changes in the immune cell phenotype, particularly macrophages, may well play a role in damage-induced chronic kidney injury.

## 5. Impact of the Adenosinergic Signaling Pathway in Radiation-Induced Normal Tissue Toxicity

A causal link between chronic accumulation of adenosine, increased extracellular matrix deposition and fibrosis development had been nicely demonstrated in the skin and the lungs of mice with genetic deficiency of ADA as well as in the models using the DNA damaging drug bleomycin (BLM) [142,143,211,212,213,214]; for a review see [215,216]. ADA-deficient mice are characterized by chronically enhanced tissue adenosine levels and collagen content as well as increased levels of pro-fibrotic mediators like TGF-β, α-SMA, CTGF and IL-13, and are therefore used as paradigm of adenosine-induced fibrosis in the lung and the skin. As mentioned above CD73 is expressed on several cell types, including cells that are responsible for matrix deposition like fibroblasts. Moreover, distinct subpopulations of fibroblasts express CD73. Nevertheless, fibroblasts and fibrocytes show differential expression of CD73; since CD73 is also a mesenchymal stem/stromal cell (MSC) marker present on fibroblast-like stromal cells it is difficult to identify and characterize these cells [217,218]. However, there is some evidence that CD73 expression levels may differ for the same cell type between the diverse tissues, e.g., for epithelial cells. CD73 is thought to be a key component of a protective pathway in epithelial barriers and critical to maintain epithelial barrier function [219]. For example, CD73 is expressed in epithelial cells of the gastrointestinal tract, the lung, the breast, the skin and the pancreas [220,221,222]. Herein, Thompson and coworkers demonstrated that the colonic intestinal epithelium expresses CD73 at higher levels than any other tissue, and that CD73 expression is limited to the apical surface of intestinal epithelial cells [118,220]. Moreover, there are differences in CD73 activity across different tissues, that can be ranked (highest to lowest) as follows: colon > kidney = brain > liver > lung > heart ≫ muscle [81].

Vice-versa, the different tissues seem to vary with respect to the cell types and adenosine receptors that are primarily responsible for driving radiation-induced pathologies [215]: Shaikh and Cronstein nicely summarized the role of ADORA2A and ADORA2B in wound healing and fibrosis [216]. For example, ADORA2A had been identified as the major mediator of the pathologic action of adenosine in the skin [223,224,225]. In contrast, the pathogenic role of adenosine in chronic pulmonary and kidney disease had mostly been linked to ADORA2B, at least in preclinical murine models [143,226,227,228]. Moreover, an association between ADORA2B and chronic pulmonary/kidney disease was also found in patients [228,229]. Among the cells that are associated with the development of fibrosis, ADORs are widely expressed on fibroblasts and epithelial cells. Briefly in a murine dermal fibrosis model ADORA2A stimulated fibroblasts to produce collagens and to downregulate matrix metalloproteinases [230]. A study with rat cardiac fibroblast revealed expression of all ADORs on these cells [231], but only ADORA2A and ADORA2B appeared to be functionally relevant. Interestingly, using ADORA2A agonist or overexpression of ADORA2B in these cardiac fibroblasts basal collagen and protein synthesis were significantly decreased, highlighting an important role of ADOR signaling in regulating cardiac fibroblast collagen synthesis [231]. Regarding the expression of adenosine receptors on epithelial cells a study from Factor et al. revealed that epithelial cells type I and type II from murine lungs both express ADORA1 and ADORA2A. Of interest, the author demonstrated that both ADORA1 and ADORA2A were expressed 3-fold in apical membranes (towards the alveolar lumen) as compared with basolateral membranes of mouse lungs. The authors suggest that apical ADOR signaling regulates alveolar fluid clearance by adenosine in a paracrine/autocrine manner [232]. Nevertheless, the role of adenosine receptors on epithelial cells in fibrosis-associated disorders and radiation-induced normal tissue toxicity is still largely unclear, though the presence of ADORs on lung epithelial cells demonstrates a potential contribution.

ATP and adenosine are also tightly regulated in the CNS between neurons and astrocytes and are important for the regulation of the neuronal network [233]. ATP functions as a neurotransmitter and exerts largely excitatory effects whereas adenosine has a major regulatory, inhibitory function within the neuronal network [233,234]. In the context of the brain adenosine mostly exerts protective functions by regulating homeostatic and metabolic networks [233,235]. Under basal conditions availability of adenosine is tightly regulated by ADK [236]. While dynamic regulation of ADK gene expression is important to early postnatal brain development, loss or inhibition of ADK promotes pathologies in the brain, e.g., cancer and epilepsy [236,237,238,239,240]. It has been demonstrated that expression changes of ADK in response to brain injury and chemotherapy-induced neuropathic pain impact the levels of protective adenosine and thus disease severity [240,241,242].

Studies about radiation-induced tissue fibrosis analyzing the influence of the adenosinergic signaling in disease pathogenesis are rare. Nevertheless, some models exist and the results are summarized in the following paragraphs.

### 5.1. CD73 and Radiation-Induced Lung-Disease

So far, the role of CD73 and adenosine in RILD has only been studied in our laboratory. In our hands, loss of CD73/adenosine signaling protected mice exposed to a single high dose irradiation with 15 Gy from RT-induced early dysfunction of the blood/air barrier without affecting the infiltration of leukocytes. Furthermore, we demonstrated that CD45^+^ leukocytes and CD45^−^ resident lung cells express CD73; nevertheless, CD73 expression was enhanced in CD45^+^ leukocytes (CD4^+^ T cells including T_reg_, alveolar macrophages) particularly during the fibrotic phase and was associated with a progressive increase in adenosine levels in the bronchioalveolar lavage fluid. In CD45^−^ resident lung cells CD73 expression was elevated particularly during the acute phase, indicating that adenosine conversion could be related to both, immune cells and to some extent to non-immune cells [74]. Definitely, further work is required to define the role of CD73 and of the disease-promoting ADORs on resident cells, immune cells or both for the tissue-, injury- and disease stage–dependent beneficial or adverse effects of adenosine in order to define biomarkers and optimal therapeutic targets. Genetic loss or pharmacologic inhibition of chronic accumulation of adenosine strongly attenuated fibrosis levels [74]. This was in contrast to findings obtained by others in preclinical models studying the role of the CD73/adenosine system in pneumonitis and transient fibrosis induced by acute intratracheal injection of bleomycin in mice. In this acute damage model, loss of CD73 in CD73^−/−^ mice exacerbated bleomycin-induced pneumonitis and fibrosis, revealing protective roles of CD73 and extracellular adenosine [142]. As opposed to this, inhibition of the low-affinity ADORA2B receptor attenuated pulmonary fibrosis induced by chronic treatment with bleomycin by repeated intraperitoneal injections [143,227] which is consistent with our findings [74,243]. Altogether these observations suggest that the beneficial or disease-promoting effects of adenosine vary depending on the tissue, the type of injury and acute vs. chronic disease stages and may be dictated by the local expression of CD73, specific adenosine receptors, or both and this might be related to local levels of tissue hypoxia [126,143,215,244].

So far, the adenosine receptor responsible for mediating disease-promoting adenosine effects in radiation-induced pulmonary fibrosis remains elusive. In own unpublished work the expression of ADORA2B was substantially higher in the un-irradiated normal lung tissue when compared to the expression levels of ADORA1, ADORA2A, and ADORA3. Furthermore, thoracic irradiation led to a significant increase in the expression of ADORA2B in C57BL/6 mice when tissue levels were determined during the fibrotic phase; instead the expression level of ADORA2B was not increased in the lung tissue of irradiated CD73 knockout mice [243]. The late appearance of the adverse adenosine effects could be due to the facts that (i) the ADORA2B is a low-affinity receptor so that higher adenosine concentrations are needed to induce significant signaling via ADORA2B and (ii) that the adenosine levels were only significantly increased as of 16 weeks after irradiation so that the pathogenic effects may only be initiated at this late time-point. 

We propose that activation of CD73/adenosine signaling amplifies pro-fibrotic signaling in the irradiated lung environment ([243] and see below) Moreover, in the model of radiation-induced pneumopathy the adverse chronic effects of CD73 and adenosine seem to predominate and may explain that treatment with inhibitory CD73 antibodies or inhibitors of adenosine signaling were beneficial [74].

### 5.2. CD73 and Radiation-Induced Skin Disease

So far, most data on the role of CD73 in dermal fibrosis originate from studies using the radiomimetic drug bleomycin or mice deficient in the adenosine-degrading enzyme ADA as follows: Loss of CD39 and/or CD73 or inhibition of ATP-release into the extracellular space strongly reduced or even prevented the development of bleomycin-induced skin fibrosis [224,245]. In line with these findings, mice deficient for ADORA2A or treated with an ADORA2A antagonist were protected from bleomycin-induced dermal fibrosis and had reduced numbers of fibrocytes in the fibrotic tissue [230,246]. Signaling via ADORA2A was recently linked to activation of the Wnt/β-catenin pathway—a known contributor to tissue fibrosis [247]. Of note, pharmacologic blockade of the ADORA2A also reduced pro-fibrotic mediators in ADA-deficient mice with chronically enhanced tissue adenosine levels and protected them from skin fibrosis [223]. Others observed a pathogenic role of ADORA2B in progression of adenosine-driven skin fibrosis [248]: ADORA2B expression was increased in fibrotic skin samples; pharmacologic blockade of ADORA2B attenuated bleomycin-induced skin fibrosis and this was associated with reduced levels of fibronectin, α-SMA^+^ myofibroblasts, hyaluronic acid and alternatively activated macrophages [248].

To our best knowledge there is only one study investigating the role of adenosinergic signaling in radiation-induced dermal fibrosis. In this model inhibition or deletion of ADORA2A attenuated dermal fibrosis development upon single high dose irradiation (40 Gy) and this was associated with reduced infiltration of T-cells, a typical feature of radiation-induced skin injury [75]. In contrast to our observations in radiation-induced pulmonary fibrosis, macrophages did not play a pathogenic role in this model, highlighting tissue-specific effects of CD73, adenosine and specific immune cells in the irradiated tissue environment with impact on radiation-induced adverse late effects.

### 5.3. Gut

So far, potential beneficial or adverse effects of CD73 and adenosine on the adverse effects of RT in the gut have not yet been studied. However, some reports point to tissue-protective effects of CD73/adenosine signaling in acute inflammatory diseases of the gastrointestinal tract by suppressing pro-inflammatory processes [79,249,250]. This is reminiscent of the above-mentioned tissue-protective effects of CD73 and adenosine in acute bleomycin-induced lung injury [142] and other models of acute tissue damage [81,118,251,252]. However these beneficial effects of CD73/adenosine signaling in the gastrointestinal tract were not only observed in diseases associated with acute inflammation in the gut but also in chronic colitis: For example, treatment of rabbits with an ADORA2A agonist significantly reduced inflammation in acute inflammatory bowel disease and further suppressed inflammatory infiltration in chronic colitis, thus preventing mortality [253]. Moreover, these beneficial effects were also induced by ADORA3 activation and led to inhibition of DSS-induced colitis in mice [254]. This protective effect of adenosine receptors in gastrointestinal inflammation was also revealed using adenosine receptor knockout mice [249,255].

These findings suggest that CD73 and adenosine have a protective function in both, acute and chronic inflammatory disease in the gastrointestinal tract, at least in a murine model of Crohns disease.

### 5.4. Kidney

Several studies investigating acute renal damage reported protective effects of CD73 and adenosine [256,257,258,259]. Furthermore ADORA2A on bone marrow derived cells protected mice from acute ischemia/reperfusion damage [260]. On the other hand, CD73 and ADORA2B were upregulated in patients and murine studies of chronic kidney injury. Here, CD73 contributed to disease progression through exaggerated production of extracellular adenosine [228].

Again, to our best knowledge there are no published data available investigating the role of the adenosinergic signaling in radiation-induced renal injury.

### 5.5. CNS

Adenosine is an endogenous neuroprotectant and modulator of cognition, and dysregulation of adenosine has been linked to radiation-induced cognitive dysfunction [238,239]. Astrocytes control the availability of adenosine though ADK-dependent metabolic clearance. Immunohistochemical analyses in rats demonstrated that exposure to cranial irradiation with 10 Gy triggered activation of ADK in the hippocampus and this was associated with astrogliosis and increased expression of glial fibrillary acidic protein (GFAP). Treatment with a pharmacologic inhibitor of ADK for 6 days prior to cranial irradiation significantly improved behavioral performance of the animals at 1 month post exposure and this was associated with reduced levels of radiation-induced astrogliosis and ADK immunoreactivity in the hippocampus [238,239]. The authors concluded that extracellular adenosine exerts neuroprotection against radiation-induced pathology and other neurological comorbidities [239,261]. Despite the important role of adenosine in the regulation of brain homeostasis and radioprotection so far, the role of CD73 in acute and chronic brain injury has not yet been investigated in detail. In studies about multiple sclerosis it was shown that postmortem samples of the brain from multiple sclerosis patients expressed high levels of CD73 in the microvasculature; moreover enhanced levels of soluble serum CD73 activity and skin microvascular CD73 expression were observed after treatment with IFN-β [262,263]. It is suggested that CD73 upregulation and subsequent adenosine production might contribute to the protective effects of IFN-β therapy in multiple sclerosis patient through improvement of the endothelial barrier function [262].

## 6. Conclusions

RT-induced direct or indirect cell death of resident cells, the resulting systemic responses to the local damage, e.g., tissue inflammation, hypoxia, vascular remodeling, and the accumulation of immune cells with disease-promoting phenotypes, contribute to the adverse late effects of RT, particularly chronic progressive pathologies such as tissue fibrosis. As outlined above, adenosinergic signaling orchestrates inflammation and repair processes in various tissues to maintain or reconstitute tissue homeostasis. However, CD73 and adenosine play dual roles in acute damage models, and chronic progressive pathologies. The role of adenosinergic signaling in fibrotic disease has been studied in preclinical models in various tissues using ADA-deficient mice or treatment with bleomycin or exposure to ionizing radiation, respectively. These investigations revealed that chronic activation of CD73/adenosine signaling mostly plays a pathologic role in fibrotic disease, as demonstrated in the lung, the skin and liver [74,75,141,143,243,264]. This assumption is corroborated by reports from ADA-deficient patients: These studies demonstrate that ADA-deficient patients develop renal complications (sclerosis) as well as pulmonary complications (pneumonitis, pulmonary alveolar proteinosis, fibrosis) [265,266,267]. Moreover, adenosine levels are also increased in patients with chronic obstructive pulmonary disease [268]. Despite the protective role of the high affinity adenosine receptor ADORA2A in acute disease models [253,260] ADORA2A also contributed to the pathologic actions of high adenosine concentrations in dermal fibrosis [75,230,246,247]. Instead, ADORA2B has been identified as a major pathologic driver in models of chronic lung disease [143,226,227,228]. 

**Figure 2:**
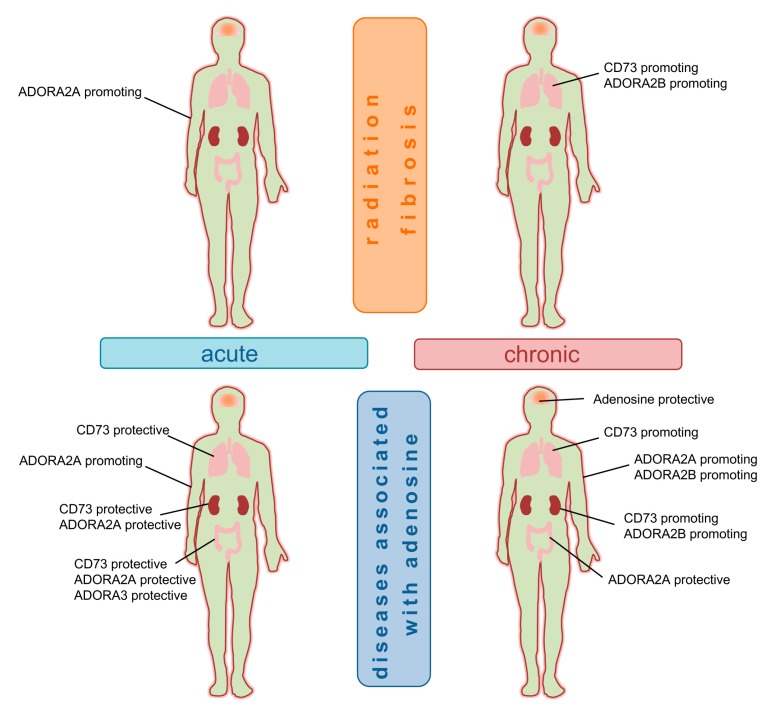
Role of Purinergic Signaling in acute and chronic disease states. Schematic overview about the different roles of components of the purinergic signaling system in acute versus chronic as well as radiation-induced versus adenosine-induced. The role of the different components is highly tissue- and model-dependent so that no general conclusions can be drawn, although there is a tendency for CD73 being a protector in acute injury models and a disease promotor in chronic injury models as well as for ADORA2B having pathologic functions in chronic disease models. (The human silhouette is only representative for a corpus. Results are obtained from clinical and preclinical data).

Figure 2 summarizes our current view on the role of adenosinergic signaling in acute and chronic adverse late effects in the lung, the skin, the kidney, the CNS and the gut presented in this review that are induced either by exposure to ionizing radiation, bleomycin or genetically-induced high adenosine levels.

In our hands, the pathogenic role of CD73/adenosine signaling in radiation-induced pulmonary fibrosis could be attributed amongst others to their ability to promote accumulation of alternatively activated pathologic macrophages in the irradiated lung tissue [243]. Interfering with signals driving the recruitment and/or alternative activation of macrophages in mice lacking CCR2 or by treatment with mAB against CSF-1R or CTGF attenuated radiation-induced lung fibrosis in mice [69,180,269,270], corroborating the pathologic role of alternatively activated macrophages in the development and progression of RILF or radiation-induced renal fibrosis, respectively [270,271]. The accumulation of macrophages expressing markers of alternative activation seems to be a common feature of several fibrotic diseases, e.g., IR-induced pulmonary fibrosis, bleomycin-induced pulmonary and dermal fibrosis, and idiopathic pulmonary fibrosis [96,226,243,248,270,272,273,274]. However their role in the disease pathogenesis remains controversial: While macrophages expressing M2-like markers could be linked to disease outcome in radiation-induced renal and lung fibrosis [243,270,271], a contribution of macrophages [275,276] or the accumulation of T cells and no role for macrophages [75] had been observed in radiation-induced dermal fibrosis. Herein it is highly likely that on myeloid cells ADORA2B may be a major pathologic driver [226].

Influx of T cells is another common phenomenon in irradiated tissues including the lung and the skin [68,75,157,277] (for a review see [278]). Adenosine signaling via ADORA2A on T_reg_ promoted T_reg_ development and their immunosuppressive function [279]; this suggests that the elevated levels of T_reg_ observed during the pneumonitic and fibrotic phase might be due to high extracellular adenosine concentrations [74,280]. T_regs_ are induced by TGF-β [281] and this cytokine is found early after lung injury as well as during the fibrotic phase so that their appearance could well be related to the induction of this pro-fibrotic mediator [282,283,284]. Vice-versa, T_reg_ secrete TGF-β, a mechanism by which they could drive fibrotic actions of other cells [285,286]. Moreover TGF-β modulates CD73/adenosine signaling directly: TGF-β induced CD73 expression on murine CD4^+^ and CD8^+^ T cells even in the absence of Foxp3 leading to adenosine-generation and immunosuppression [287,288]. While the presence of T_reg_ aggravated BLM-induced lung fibrosis in mice [289] T_reg_-depletion with an anti-CD25 antibody inhibited fibrocyte recruitment and reduced radiation-induced pulmonary fibrosis [290].

Altogether these observations highlight a functional relevance of M2-like macrophages, T_reg_, or both, in fibrotic disease induced by chronic adenosine accumulation, at least in mice. Of note, a recent report highlighted a switch towards fibrosis-associated M2-like macrophage/T-helper cell type 2-like polarized phenotypes also in mice exposed to a clinically more relevant fractionated WTI [46].

The differences in the role of CD73/adenosine signaling in different pathologies presented here strongly suggest a contribution of differences in the characteristics of the tissues investigated [215]. In this context, differences in the tissue environment such as extracellular matrix composition, immune cell repertoire, expression of components of the CD73/adenosine signaling pathway on resident cells, recruited immune cells or fibroblasts (e.g., adenosine receptors), or the expression of CD73/adenosine-modulating signaling molecules may play a role as discussed in detail elsewhere [216]. Furthermore, differences in the radiosensitivity of specific resident cells or resident immune cells, or in the behavior of irradiated tissue stem cells may also play a role [243,291,292,293,294]. Finally, these differences might also be due to tissue-specific expression patterns of fibrosis-modulating signaling molecules in resident cells or recruited immune cells, tissue-specific damage responses, or both [215,216].

For example, the data obtained so far suggest that ADORA2B may play a more important pathogenic role than ADORA2A in the pathogenesis of radiation-induced pulmonary fibrosis, BLM-induced pulmonary fibrosis, idiopathic pulmonary fibrosis and chronic kidney disease, whereas ADORA2A seems to be a major player in radiation- and BLM-induced dermal fibrosis. It is thus tempting to speculate that ADORs have more comparable functions in models of radiation-induced tissue fibrosis in lung, gut and kidneys, and that their functions in the regulation of the immune environment of these tissues might be distinct of those in the skin. Interestingly, new findings about disease-promoting functions of ADORA2B in dermal fibrosis [248] indicate an even more complex network between disease-promoting and protective functions of the ADORs in acute and chronic injury models, which also depend on the tissue investigated.

However, it has also to be considered that the models of radiation-induced fibrosis in the lung, gut, kidney and skin are characterized by substantial differences with respect to the radiation dose (15 Gy in the lung, 12–18 Gy in the intestine, 8–10 Gy WBI for kidney injury versus 35–40 Gy in the skin) and the time course (weeks versus months). Thus, some of the differences in the findings about the roles of ADORA2A and ADORA2B in radiation-induced dermal and pulmonary fibrosis may well be due to the differences in the damage model [42].

However, it remains to be investigated if such distinct functions are due to differences in the tissue environment, or can at least in part be attributed to the differences in the radiation dose as skin fibrosis was initiated by applying a single high dose to the organ whereas the models for the other tissues use rather intermediate radiation doses.

## 7. Outlook—Clinical Translational Perspectives

Clinical RT aims to expose the tumor volume to the maximal dose for reducing tumor burden while sparing normal tissues to minimize the risk for long-term injury. However, adverse late effects of IR in normal tissues still limit successful RT.

To overcome this limitation radiobiologists, focus on developing innovative biological strategies that are designed to interfere selectively with the pathologic processes driving RT-induced adverse late effects without protecting the tumor and thereby can prevent, attenuate, or reverse acute and late RT-induced normal tissue toxicities. Such treatments would be suited to improve the quality of life of irradiated patients, or allow for treatment intensification efforts at least under certain conditions [3,7].

As outlined in this review chronic activation of CD73/adenosine signaling mostly plays a pathogenic role in chronic radiation-induced adverse late effects and thus constitutes an attractive therapeutic target for attenuating the adverse late effects of RT. However, the therapeutic ratio can also be improved when RT is combined with chemotherapy or molecularly targeted drugs that are suited to radiosensitize the tumor cells, to overcome resistance-promoting signals in the tumor environment, or both [5,23]. Research in radiation biology has therefore also a strong focus on defining innovative biological concepts to specifically increase the radiosensitivity of the tumor tissue without enhancing the risk of normal tissue complications. This may also allow to reduce the RT dose without reducing treatment efficacy.

An optimal strategy for increasing the therapeutic ratio would thus sensitize the tumor to the cytotoxic effects of IR and at the same time protect normal tissues from its adverse late effects. As nicely highlighted in recent reviews, extracellular adenosine is also generated at high levels in the microenvironment of solid tumors, particularly in the context of hypoxia [126,295,296,297,298]. High extracellular adenosine levels in the tumor microenvironment play a critical role in tumor progression on the one hand by direct stimulation of tumor cell proliferation, migration, invasion, metastatic dissemination [299,300] and on the other hand by potent suppression of antitumor immunity (for more details see below). Furthermore, first studies in patient samples point to a correlation between high CD73 expression and poor prognosis in cancer, e.g., in non-small cell lung cancer (NSCLC), triple negative breast cancer, high grade serous ovarian cancer, head and neck squamous cell carcinoma (HNSCC) [301,302,303,304,305].

In more detail, release of ATP through exocytosis or Pannexin-1 channels and subsequent CD39/CD73-dependent extracellular degradation to adenosine contribute to the accumulation of non-physiologically high levels of adenosine in the tumor microenvironment, particularly in hypoxic tumors that may reach extracellular concentrations in the µM range (0.2 to 100 μM) compared to 10–300 nM in normal tissues [74,121,122,126,127]. Various preclinical studies demonstrate that expression of CD73 on tumor cells and the resulting adenosine generation and signaling directly support tumor cell proliferation and neovascularization, as well as tumor metastasis [306,307,308,309,310]. For example, genetic loss or pharmacologic inhibition of CD73 as well as inhibition of ADORA2B reduced the metastatic potential of tumor cells in preclinical studies [306,309,311,312,313,314]. Patient studies confirmed increased levels of CD73 in metastatic tumors [315,316]. Interestingly, high levels of CD73 have been associated with resistance to HER2/ErbB2 inhibitor therapy in preclinical breast cancer models and poor clinical outcome of breast cancer patients under Trastuzumab therapy [305]. Moreover, CD73 was induced during T-cell immunotherapy in a murine melanoma in relapsed tumors which acquired a mesenchymal-like phenotype [317]. In line with these preclinical investigations the authors of this study also detected up-regulation of CD73 in melanoma patients progressing under adoptive T-cell transfer or immune checkpoint blockade [317]. These observations point to an important role of CD73 and adenosine signaling as a mechanism of intrinsic or adaptive resistance that may be amenable to therapeutic targeting.

Importantly, CD73 and adenosine also act as modulators of tumor immunity with potent immunosuppressive actions and thereby support tumor immune escape [304,308,310,318,319,320,321,322,323]. The immunomodulatory actions of CD73 and adenosine and their contribution to tumor immune escape include for example inhibition of CD4^+^ and CD8^+^ T cells, natural killer cells and antigen-presenting dendritic cells as well as the induction of immunosuppressive cells such as T_reg_, myeloid-derived suppressor cells (MDSCs), and macrophages with alternative M2 like activation state that have been extensively summarized elsewhere [103,323,324,325,326] and will therefore not be discussed in more detail here. Pharmacologic inhibition of CD73, ADORA2A, or both, relieved adenosine-mediated immunosuppression, improved antitumor immune responses and treatment outcome when given alone or in combination with other immunotherapies in preclinical investigations [325,327,328,329,330,331]. Of note, inhibition of CD73 by a clinically relevant inhibitory antibody (MEDI9447) significantly altered both, myeloid and lymphoid cells in the microenvironment of syngeneic murine tumors; these changes involved increases in CD8^+^ effector cells and activated macrophages and correlated with responding compared to non-responding tumors [329] which is reminiscent of our findings in the irradiated murine lung [74,243]. Importantly, CD73-inhibition not only enhanced control of local tumors but also of tumor metastases, and revealed increased efficacy in combination with anti-PD-1 antibodies [329,332].

In our hands, exposure of mice to a single high dose whole thorax irradiation lead to a time-dependent induction of CD73 expression and activity in non-hematopoietic and hematopoietic host cells. Furthermore, chronic activation of CD73 and accumulation of adenosine were critical to the phenotypic switch of infiltrating innate and adaptive immune cells towards immunosuppressive cell types [74,243]. It is thus highly likely that RT-induced damage might also trigger release of adenosine, activation of CD73, or both, in the irradiated tumor microenvironment; hypoxia-induced or RT-induced increase in extracellular adenosine levels might then limit RT-induced immune enhancement and increase resistance to combined radioimmunotherapy by adenosine-mediated immunosuppression [42,72,244,295,333]. Intriguingly, circulating CD4^+^ T cells and CD4^+^CD25^hi^ T_reg_ of HNSCC patients including patients that had received RT as part of multimodal treatment displayed up-regulated expression of CD39 and hydrolyzed ATP at higher rates and produced higher levels of adenosine than normal controls’ T_reg_. This points to increased activity of CD73 and may further dampen anti-tumor immune responses in irradiated patients [104]. Nevertheless the role of CD73/adenosine signaling in human T_reg_ needs to be further elucidated since T_reg_ show mainly enriched intracellular CD73 expression but remain negative for CD73 surface expression. Thus, human T_reg_ need to cooperate with another subset of cells, expressing surface-bound CD73, to favor adenosine-mediated immune suppression, and this could largely impact the use of new therapeutic drugs targeting the CD73/adenosine pathway.

We thus propose that the CD73/adenosine signaling is an attractive therapeutic target for improving the therapeutic gain of RT: we expect that pharmacologic inhibition of CD73/adenosine signaling will not only protect irradiated tissues from early and late adverse side effects of irradiation but block at the same time tumor-promoting effects of CD73/adenosine signaling in the tumor environment and overcome CD73/adenosine-mediated tumor immune escape [42,244]. As mentioned before the expression pattern of CD39 and CD73 on immune cells (T_reg_, CD4^+^ T cells, CD8^+^ T cells, B cells and macrophages) differs between mice and humans. These differences might impact the role of CD73/adenosine signaling in irradiated normal and tumor tissues and impede translation into the clinical situation. Thus, further work is required to validate the role of the CD73/adenosine pathway in patient samples, particularly with respect to normal tissues. That the CD73/adenosine pathway might be clinical relevant in normal tissue toxicities comes from existing data of COPD and IPF patients demonstrating that the CD73/adenosine pathway and A2BR signaling promotes inflammation and profibrotic mediators in the lung [96]. Moreover, the efficacy of targeting CD73/adenosine signaling in human pathologies needs to be (further) elucidated.

Several therapeutic options exist to target adverse CD73/adenosine signaling: (i) avoid excess extracellular adenosine accumulation by inhibitory CD73 antibodies [74,313,322]; (ii) degrade excess adenosine by application of PEG-ADA [74] which is already successfully used since years in the treatment of ADA deficiency in patients [334]; (iii) impair pathologic actions of adenosine by inhibitors of disease-promoting ADORs [138], respectively. The hypoxia-induced, hypoxia-inducible factor-1 alpha (HIF-1α)-dependent expression of CD73 and ADORAs in tumor cells and host cells [219,320,335,336,337] make CD73 and adenosine promising targets particularly for tumors with pronounced hypoxia, high HIF-1α–expression, or both, (e.g., [244,295]).

## 8. Final Remarks

The biology-based optimization of RT requires the identification of biological factors that are linked to the radiosensitivity of normal tissues or tumor tissues and that are amenable to therapeutic targeting. To our view, the adenosinergic signaling pathway offers multiple possibilities to improve RT outcome: (i) protecting the normal tissue from adverse late effects like tissue fibrosis, (ii) sensitizing the tumor tissue for radiotherapy by relieving intrinsic or hypoxia-driven adenosinergic immune suppression, and (iii) improving tumor control by reducing angiogenesis and metastatic potential. Successful translation of such approaches into the clinic will largely depend on the definition of reliable biomarkers that predict individual radiosensitivity in patients. In the era of “multi-omics” research focusses on the systematic analysis of individual gene, miRNA or protein expression profiles in irradiated tumor and normal tissues of individual patients in order to define expression signatures predictive for the radiosensitivity of tumor and normal tissues [5]. Genomic interrogation for biomarkers of tumor radiosensitivity and normal tissue toxicity such as the CD73/adenosine pathway might well be suited to gain new mechanistic insight into tissue-specific roles of specific immune cell types from the innate and adaptive immune system, and disease-promoting aspects of the multifaceted cross-talk between damaged resident cells, extracellular matrix molecules, soluble mediators and infiltrating immune cells including tissue-specific activation of signaling pathways in the irradiated tissue environment. The identification of such predictive profiles will facilitate the selection of the individual RT dose and of appropriate combination strategies for improving the therapeutic ratio in the future by targeting immunomodulatory pathways such as the CD73/adenosine pathway.
