# Addendum: de Leve, S.; et al. The CD73/Ado System—A New Player in RT Induced Adverse Late Effects. *Cancers* 2019, *11*, 1578

**DOI:** 10.3390/cancers11121898

**Published:** 2019-11-28

**Authors:** Simone de Leve, Florian Wirsdörfer, Verena Jendrossek

**Affiliations:** Institute of Cell Biology (Cancer Research), University Hospital Essen, 45122 Essen, Germany; simone.deleve@uk-essen.de (S.d.L.); florian.wirsdoerfer@uk-essen.de (F.W.)

The authors would like to make an addendum to their published paper [1].

There was a missing annotation and reference in the original version of the article in the caption of Figure 1 in [1]:

The authors wish to mention that some elements from Figure 1 are inspired from Salmi and Jalkanen [2] and modified from Cappuccini, “Radiation-induced pneumonitis and fibrosis—Defining the role of immune cells and regulatory molecules” (Ph.D. thesis) [3].

The changes do not affect the scientific results.

The rest of the manuscript does not need to be changed. The authors would like to apologize for any inconvenience caused. 
